# Glycoproteoform Profiles of Individual Patients’ Plasma Alpha-1-Antichymotrypsin are Unique and Extensively Remodeled Following a Septic Episode

**DOI:** 10.3389/fimmu.2020.608466

**Published:** 2021-01-14

**Authors:** Tomislav Čaval, Yu-Hsien Lin, Meri Varkila, Karli R. Reiding, Marc J. M. Bonten, Olaf L. Cremer, Vojtech Franc, Albert J. R. Heck

**Affiliations:** ^1^ Biomolecular Mass Spectrometry and Proteomics, Bijvoet Center for Biomolecular Research and Utrecht Institute for Pharmaceutical Sciences, University of Utrecht, Utrecht, Netherlands; ^2^ Netherlands Proteomics Center, Utrecht, Netherlands; ^3^ Department of Intensive Care Medicine, University Medical Center Utrecht, Utrecht, Netherlands; ^4^ Julius Center for Health Sciences and Primary Care, University Medical Center Utrecht, Utrecht, Netherlands; ^5^ Department of Medical Microbiology, University Medical Center Utrecht, Utrecht, Netherlands

**Keywords:** acute-phase proteins, sepsis-diagnostics, glycoproteomic analysis, alpha-1-antichymotrypsin, acute phase response (APR)

## Abstract

Sepsis and septic shock remain the leading causes of death in intensive care units (ICUs), yet the pathogenesis originating from the inflammatory response during sepsis remains ambiguous. Acute-phase proteins are typically highly glycosylated, and the nature of the glycans have been linked to the incidence and severity of such inflammatory responses. To further build upon these findings we here monitored, the longitudinal changes in the plasma proteome and, in molecular detail, glycoproteoform profiles of alpha-1-antichymotrypsin (AACT) extracted from plasma of ten individual septic patients. For each patient we included four different time-points, including post-operative (before sepsis) and following discharge from the ICU. We isolated AACT from plasma depleted for albumin, IgG and serotransferrin and used high-resolution native mass spectrometry to qualitatively and quantitatively monitor the multifaceted glycan microheterogeneity of desialylated AACT, which allowed us to monitor how changes in the glycoproteoform profiles reflected the patient’s physiological state. Although we observed a general trend in the remodeling of the AACT glycoproteoform profiles, e.g. increased fucosylation and branching/LacNAc elongation, each patient exhibited unique features and responses, providing a resilient proof-of-concept for the importance of personalized longitudinal glycoproteoform profiling. Importantly, we observed that the AACT glycoproteoform changes induced by sepsis did not readily subside after discharge from ICU.

## Introduction

Sepsis, a life-threatening bloodstream infection, is a major cause of morbidity and mortality in hospitals worldwide, especially at their Intensive Care Units (ICUs), occurring in approximately 3–5 people per 1,000 population half of which require ICU treatment (1, 2). Mortality rates reach up to 50% for patients experiencing septic shock (1–5). Diagnosis is currently largely based on symptom scores and microbiological data, which lack both sensitivity and specificity and can lead to significant delays in the initiation of adequate treatment. To date, no single biomarker has been able to diagnose sepsis in an efficient and timely manner. Therefore, further research remains imperative to improve our understanding and knowledge of sepsis and develop new diagnostics and prognostics tools (6).

Of patients admitted to the hospital, blood, plasma, and serum are readily accessible and represent a rich source of proteins used in clinical diagnostics. Consequently, over the last two decades, hundreds of studies focused on mining plasma or serum for biomarkers of all kinds of diseases, notably cancer, diabetes, sepsis, and other inflammatory or infectious diseases (7). Recently, due to the improvements in sample preparations and mass spectrometry-based proteomics technologies, we have been witnessing a renewed interest in plasma proteomics (7, 8). One notable example is a recent large scale study of plasma protein levels in people during longitudinal weight loss, where almost half of all monitored proteins had individual-specific levels that varied significantly between individuals but stayed relatively constant in abundance within each individual (9). Related to sepsis, a vast body of investigations has hinted at close to two hundred proteins associated with a sepsis response (6, 10–15), with the general pathways represented by these proteins being acute-phase response (APR), complement activation, and blood coagulation. Unfortunately, many of these proteins are also putative biomarkers for several other pathophysiological conditions, and thus not unique for sepsis (6).

During the APR, the concentration in plasma of several acute-phase proteins (APPs) changes, which is a part of a very complex systemic response to many diseases. Among the most important APPs is C-reactive protein (CRP) that consequently is frequently used in diagnostics as a marker of inflammation and infection (16–18). Measuring CRP values has proven to be very useful in determining treatment effectivity or progress of disease/recovery (19).

In addition to changes in protein plasma levels, variations can also occur at a post-translational level, notably in protein glycosylation. These alterations can be specific and are highly dependent on the pathophysiological processes occurring in the cell/tissue producing the protein, and on down-stream processing when the protein is secreted into the bloodstream (20–23). As glycosylation is ubiquitous in proteins found in extracellular environments, the study of glycosylation patterns in proteins from body fluids, such as plasma, has become an important source of information to describe cellular processes, including acute inflammation during infection. However, the inherent vast structural complexity of glycoproteins and their proteoform profiles represent a notorious analytical challenge. Moreover, plasma protein glycosylation is regulated by various factors such as the genetic background of the donor and their environment, making each patient likely unique (24, 25). We recently reported evidence for this uniqueness, reporting that the proteoform profiles of two abundant plasma APPs, fetuin and haptoglobin, exhibit patient-specific attributes, some of them caused by genetic polymorphisms (26, 27). These observations underline the urgency to investigate plasma proteins in patients individually, i.e. personalized diagnostics not only at the protein but also proteoform level, as we here aim to present for α-1-antichymotrypsin (AACT).

Despite many obstacles in identifying specific biomarkers for sepsis, a study from DeCoux et al. has demonstrated an increased abundance of specific glycopeptides, including several originating from AACT, with sepsis survivors (28). We follow up on these key discoveries and here investigate AACT (glyco)proteoforms in unprecedented molecular detail in individual donors that all experienced, and recovered from, a septic episode.

Human AACT is a fairly abundant plasma protein and member of the serpin superfamily of serine proteinase inhibitors that play a key role in the control of several proteolytic cascades. It is mainly produced by the liver and bronchial epithelial cells (29). During an inflammatory response, plasma levels of AACT can be doubled within 16 h (30), which classifies AACT among the positive-response APPs. Although AACT was described more than half a century ago (31), its precise function remains quite elusive. In general, AACT has a role as an anti-inflammatory agent, inhibiting chymotrypsin, cathepsin G, and other proteases (32, 33). AACT is a heavily glycosylated protein with an apparent molecular mass estimated by SDS-PAGE between 55,000 and 66,000 Da, with the variation attributed to glycosylation microheterogeneity. The protein contains six putative *N*-glycosylation sites, and intact glycopeptide analysis confirmed five sites as occupied (GlyGen P01011), namely asparagine residues at positions 93, 106, 127, 186, and 271 (**Supplementary Figure 1**) (34, 35). NMR analysis of healthy serum AACT released N-glycans revealed mainly di- and triantennary N-glycans (36); however, a more detailed analysis of the composition of each *N*-glycan and its heterogeneity has not been provided yet.

Here, we employed state-of-the-art high-resolution native mass spectrometry (MS) to analyze 40 plasma samples obtained from ten patients, each at four different time-points, who were initially admitted to the ICU for routine observation yet later developed sepsis. We first monitored the plasma proteome profiles from these patients across the different time points to elucidate changes in protein abundances associated with sepsis, confirming that several proteins, including CRP, AACT, and several other APPs, became higher abundant at the onset of sepsis (37–39). Next, we performed quantitative glycoproteoform profiling of AACT to monitor potential changes in response to sepsis, using high-resolution native MS (40, 41). Cumulatively, our data revealed that at the onset of sepsis a substantial glycosylation remodeling occurs in particular on AACT, whereby it becomes decorated with glycans exhibiting increased branching/LacNAc elongation and fucosylation. Although the glycan remodeling during the septic episode revealed similar features in all patients, each patient carried a distinctive, unique glycosylation signature. Notably, our data revealed that the AACT glycoproteoform changes induced by sepsis did steadily continue after discharge from ICU, even when the AACT serum concentration had gone back down to pre-sepsis levels.

## Methods

### Individual Plasma Sample Collection and Chemicals

For this study, samples were selected from an existing prospective study database of patients (Molecular Diagnosis and Risk Stratification of Sepsis—MARS cohort), who were initially admitted to the ICU for observation following elective surgery, but who developed nosocomial sepsis later during their hospital stay and were consequently re-admitted to the ICU. Median SOFA scores of the patients were 6.5 (range 6–9). Patients’ EDTA plasma was obtained during routine ICU blood collection and 2x 450 µL was stored at −80°C within 4 h after collection. CRP was analyzed in heparin plasma on an AU5811 routine chemistry analyzer (Beckman Coulter, Brea, California). The UMCU institutional review board approved an opt-out method for consent (protocol numbers 10-056C/18-192).

Unless otherwise specified, all chemicals and reagents were obtained from Sigma-Aldrich (Steinheim, Germany). Acetonitrile (ACN) was purchased from Biosolve (Valkenswaard, The Netherlands). Sequencing grade trypsin was obtained from Promega (Madison, WI). The Oasis PRiME HLB plates were purchased from Waters (Etten-Leur, the Netherlands).

### Plasma Sample Preparation—Depletion of Three Abundant Proteins

One hundred micro liter of each plasma sample (40 samples in total) was first filtered by using a 0.45 μm filter membrane and subsequently immunodepleted by using a Human plasma Depletion Gravity Column (Good Biotech Corp., Taichung, Taiwan) for the removal of albumin, IgG, and transferrin. The depletion procedure was performed according to the vendor’s protocol. Briefly, the column was first equilibrated with an 8 ml PBS buffer. Next, 100 μL of filtered plasma was pipetted on the top of the resin, followed by 1 mL PBS to yield sufficient volume for transferring the plasma through the resin. After 5 min, the collected flow through, which does not contain any proteins, was discarded. The depleted plasma was eluted from the resin with five column volumes of 1 mL PBS (in total 5 mL) and collected 5 mL PBS fraction was concentrated down to around 50 μL by using a 10 kDa cutoff filter. The concentrated plasma samples were stored at −80°C until the ion-exchange chromatography separation.

### α-1-Antichymotrypsin Purification From Individual Plasma Samples

To purify AACT an Agilent 1290 Infinity HPLC system (Agilent Technologies, Waldbronn, Germany) consisting of a refrigerated autosampler with a 500-μL injector loop, a binary pump, a vacuum degasser, two-column compartment with thermostat, auto collection fraction module, and multi-wavelength detector. The system consisted of a tandem CAT- WAX (PolyWAX LP, 200 × 2.1 mm i.d., 5 μm, 1,000 Å; PolyCAT A, 50 × 2.1 mm i.d., 5 μm, 1,000 Å) two-stage column set up. The autosampler was connected to the first column (CAT) by a 500 mm sample line (SecurityLINK PEEKsil 500 x 0.1 mm i.d.). The second column (WAX) was connected to the sample collector using a 500 mm line (SecurityLINK PEEKsil 500 x 0.1 mm i.d.). The columns were connected by a 150 mm tubing (SecurityLINK PEEKsil 150 x 0.1 mm i.d.). All connecting lines were obtained from Phenomenex B. V. (Utrecht, Netherlands). Both columns were obtained from PolyLC Inc. (Columbia, USA). The column compartment was kept at 17°C while the other compartments were cooled to 4°C. Mobile phase Buffer A consisted of 100 mM ammonium acetate (AMAC) (pH 7.2) in water, and Buffer B consisted of 2.5 M AMAC in water. Typically, 50 μL of depleted plasma sample was mixed with 250 μL Buffer A and injected per run. Elution was achieved using a multi-step gradient, consisting of six transitions with increasing proportions of Buffer B: (step 1; equilibration) 0% B, 0–5 min; (step 2; salt gradient) 0–11% B, 5–6.5 min; (step 3; salt gradient) 11–36% B, 6.5–23 min; (step 4; high salt rinse) 36–100% B, 23–27 min; (step 5; high salt wash) 100% B, 27–31 min; (step 6; restoration) 100–0% B. The flow rate was set to 800 μL/min. The chromatograms were monitored by absorption at 280 nm and time-based fractions collected every 0.5 min from 12–20 min using an automated fraction collector. Human AACT eluted in the fraction collected in between 15.50–16.00 min (**Supplementary Figure 2**).

### Native Mass Spectrometry Analysis of α-1-Antichymotrypsin

For each patient and each time-point, the tandem CAT-WAX fraction containing the AACT protein was first buffer exchanged into 150 mM AMAC (pH 7.5) and concentrated to a volume of ~50 µL by ultrafiltration (Vivaspin 500 μL, Sartorius Stedim Biotech, Germany) using a 10 kDa cut-off filter. Next, the samples were treated with sialidase (Neuraminidase from *Arthrobacter ureafaciens*) to remove sialic acids (Merck KGaA, Darmstadt, Germany). The desialylation was performed by incubating the sample with 0.02 U of sialidase 8 h at room temperature. The resulting total protein concentration was estimated by ultraviolet absorbance at 280 nm and adjusted to ~2–5 µM prior to native MS analysis. The desialylated AACT samples were subsequently analyzed on a modified Exactive Plus Orbitrap instrument with extended mass range (EMR) (Thermo Fisher Scientific, Bremen) as described previously (42). The measurements were conducted in positive mode and a standard *m/z* range of 1,000–10,000 was used (43). The voltage offsets on the transport multi-poles and ion lenses were manually tuned to achieve optimal transmission of protein ions at elevated *m/z*. Nitrogen was used in the HCD cell at a gas pressure of 6–8 × 10^−10^ bar. The previously optimized MS parameters were used (42): spray voltage 1.2–1.3 V, source fragmentation 30 V, source temperature 250°C, collision energy 30 V, and resolution (at *m/z* 200) 17,500. The mass spectrometer was calibrated using CsI clusters, as described previously (42).

The masses of the observed glycoproteoforms of AACT were extracted from the zero-charge deconvoluted native mass spectra using Intact Mass software (Protein Metrics ver. 3.8) (44). Parameters for spectra deconvolution were used as follows: min difference between mass peaks was set to 15 Da, charge vectors spacing was set to 0.4, smoothing sigma was set to 0.02 m/z, spacing was set to 0.04 m/z and peak sharpening was enabled. Other parameters were set to be default.

In the mass calculations, we used the average mass of the AACT sequence (UniPro code: P01011-1, Isoform 1) lacking the N-terminal signaling propeptide, which resulted in the mass of 45,265.82 Da. Based on AACT’s backbone mass, we created a table, including the theoretical masses of all possible AACT glycoproteoforms with different glycan compositions. The glycan composition was chosen based on known biosynthetic pathways and reported glycan structures in plasma glycomics literature. Next, we manually matched the masses of the observed glycoproteoforms of AACT with the theoretical ones within the mass tolerance of 200 ppm. The average masses used for the glycans were: hexose/mannose/galactose (Hex/Man/Gal, 162.1424 Da), *N*-acetylhexosamine/*N*-acetylglucosamine (HexNAc/GlcNAc, 203.1950 Da), deoxyhexose (dHex, 146.1430 Da). Peak intensities in the deconvolved mass spectra were used for the relative quantification of all the co-occurring AACT glycoproteoforms and correlation matrix analysis. Each proteoform was normalized so that the sum of all proteoforms in a given mass spectrum amounted to 100%. The Pearson correlation coefficient was used for the correlation matrix analysis to evaluate the similarity among the deconvoluted native spectra derived from all 10 patients with 4 time points. The deconvoluted masses of the observed glycoproteoforms of AACT were loaded into R package corrplot (version 3.5.1), and then the corresponding similarity matrix was produced based on the Pearson correlation method.

For the statistical analyses, we applied a standard repeated measure one-way-ANOVA test to determine if there is a statistically significant difference in the weighted average masses (WAM) across different time points. Post hoc t-test with Bonferroni correction was used to test the differences between each of the points.

### Glycopeptide Profiling of Isolated α-1-Antichymotrypsin

The desialylated AACT samples from Patient 10, which were previously analyzed by Native MS, were utilized for glycopeptide profiling using bottom-up MS approach to support out native MS annotations. Five micro liter of desialylated AACT protein was taken from the concentrated IEX fraction and dissolved into 45 µL digestion buffer (components of digestion buffer was described in the “plasma proteome profiling” part). Glu-C was first added for 3 h digestion at 37°C at an enzyme-to-protein ratio of 1:75 (w/w) and the resulted peptide mixtures were further digested by using trypsin (1:100; w/w). The next day SDC was removed as described in the plasma proteome profiling part), and all proteolytic digests containing modified glycopeptides were desalted using Oasis PRiME HLB plate, then dried and stored at −80°C until MS analysis.

Peptides were separated and analyzed using the HPLC system (Agilent Technologies, Waldbronn, Germany) coupled on-line to an Orbitrap Fusion Lumos mass spectrometer (Thermo Fisher Scientific, Bremen, Germany) using a 65 min gradient: 0–5 min, 100% solvent A; 13–44% solvent B for 45 min; 44–100% solvent B for 5 min; 100% solvent B for 5 min; 100% solvent A for 5 min. For the MS scan, the mass range was set from m/z 350 to 2,000 with a maximum injection time of 50 ms at a mass resolution of 120,000 and an AGC target value of 5x10^4^ in the Orbitrap mass analyzer. The dynamic exclusion was set to 30 s for an exclusion window of 10 ppm with a cycle time of 3 s. Charge-states screening was enabled, and precursors with 2^+^ to 12^+^ charge states and intensities > 1e^5^ were selected for MS^2^. HCD MS^2^ (m/z 100–2,100) acquisition was performed in the HCD cell, with the readout in the Orbitrap mass analyzer at a resolution of 30,000 (isolation window of 1.6 Th) and an AGC target value of 5x10^4^ or a maximum injection time of 75 ms with a normalized collision energy of 30%. If at least 1 out of 3 glycopeptide oxonium ions (138.0545 + ^1^, 204.0687 + ^1^, 366.1396 + ^1^) were observed, EThcD MS^2^ of the same precursor was triggered (isolation window of 1.6 Th) and fragment ions were analyzed with an extended mass range approach (m/z 100–3,000) in the Orbitrap mass analyzer at a resolution of 30,000 (45). AGC target value of 2x10^5^ or a maximum injection time of 100 ms with activation of ETD and supplemental activation with normalized collision energy (NCE) of 27%.

The raw data files containing MS^2^ spectra of AACT peptides were processed using Byonic software (version 3.4.0) (Protein Metrics Inc., United States) with the following parameters: precursor ion mass tolerance, 10 ppm; product ion mass tolerance, 20 ppm; fixed modification, Cys carbamidomethyl; variable modification: Met oxidation. We used the Byonic database of 182 glycans with no multiple fucoses for the N-glycan analysis, whereby we added several reported glycan compositions with multiple fucoses (**Supplementary Table 1**). The allowed number of miss-cleavages was set to 3. Trypsin (C-terminal RK) and Glu-C (C-terminal DE) enzyme specificity search was chosen for all samples. The fasta file used for the peptide searches contained AACT amino acid sequences (UniPro code: P01011-1, Isoform 1). Byonic peptide cut-off score of 200 was used and all PTM-modified identified spectra were further manually inspected. Site-specific quantification of the AACT N-glycosylation sites was performed as follows; the first three isotopes were taken from each manually validated peptide proteoform for the calculation of the peak areas. Each peptide that contained glycosylation sites was normalized individually so that the sum of all its proteoform areas was set to 100%. The average peptide ratios from all measurements were taken as a final estimation of the abundance. The extracted ion chromatograms (XICs) were obtained with Skyline (46). The glycan structures of each glycoform were manually annotated. Hereby, reported glycan structures are depicted without the linkage type of the glycan units as our acquired MS/MS data do not directly provide such information.

### Plasma Proteome Profiling

For an overall rough inventory of the proteome, 2 µL of each plasma sample (~75 mg/mL) was taken and mixed with 50 µL digestion buffer prior to standard reduction and alkylation of the proteins. Of note, these plasma proteome analyses were performed without the depletion of any proteins and desialylation. The digestion buffer contained 100 mM Tris–HCl (pH 8.5), 1% w/v sodium deoxycholate (SDC), 5 mM Tris (2-carboxyethyl)phosphine hydrochloride (TCEP) and 30 mM chloroacetamide (CAA). Trypsin was then added for overnight digestion at 37°C at an enzyme-to-protein ratio of 1:20 (w/w). The next day the SDC was removed *via* acid precipitation (final concentration 0.5% trifluoroacetic acid) (TFA), whereafter the peptides were desalted using an Oasis PRiME HLB plate according to the vendor’s protocol (Waters), then dried and stored at −80°C until MS analysis.

Prior LC-MS/MS analysis, the plasma samples were reconstituted in 150 μL of 1% FA and 1 μL was used for one injection. LC-MS/MS analysis on these samples was performed by coupling an Agilent 1290 Infinity HPLC system (Agilent Technologies, Waldbronn, Germany) to a Q Exactive HF-X mass spectrometer (Thermo Fisher Scientific, Bremen, Germany). The peptides were first trapped with a 100 μm inner diameter 2 cm trap column (in-house packed with ReproSil-Pur C18-AQ, 3 μm) (Dr. Maisch GmbH, Ammerbuch-Entringen, Germany) coupled to a 50 μm inner diameter 50 cm analytical column (in-house packed with Poroshell 120 EC-C18, 2.7 μm) (Agilent Technologies, Amstelveen, The Netherlands). The mobile-phase solvent A consisted of 0.1% FA in water, and the mobile-phase solvent B consisted of 0.1% FA in 80% ACN. A 115 min gradient was used as follows: 0–5 min, 100% solvent A; 13–44% solvent B for 95 min; 44–100% solvent B for 5 min; 100% solvent B for 5 min; 100% solvent A for 10 min. Peptides were ionized using a spray voltage of 1.9 kV and a heated capillary (250°C). The mass spectrometer was set to acquire full-scan MS spectra (375–1600 *m/z*) for a maximum injection time of 20 ms at a mass resolution of 60,000 and an automated gain control (AGC) target value of 3e6. Up to 15 of the most intense precursor ions were selected for tandem mass spectrometry (MS/MS). HCD MS/MS (200-2000 *m/z*) acquisition was performed in the HCD cell, with the readout in the Orbitrap mass analyzer at a resolution of 30,000 (isolation window of 1.4 Th) and an AGC target value of 1e5 or a maximum injection time of 50 ms with a normalized collision energy of 27%.

MaxQuant (software version 1.6.3.4) (47) was used to analyze the LC-MS/MS raw files. MS/MS spectra were searched against the Swiss-Prot FASTA database (release date: Feb 2018, 20 412 entries, taxonomy: *Homo sapiens*) with the following modifications: fixed cysteine carbamidomethylation, variable methionine oxidation. Enzyme specificity was set as C-terminal to arginine and lysine with a maximum of 2 missed cleavages. The searches were performed using a precursor mass tolerance of 8 ppm and a fragment mass tolerance of 20 ppm followed by a 1% false discovery rate (FDR) for protein and peptide levels with a minimum length of six amino acids for peptides. For label-free quantification, the “match between run algorithm” in the MaxQuant quantification suite was used. Label-free protein quantification (LFQ) was performed for unique peptides with a minimum ratio count > 2.

## Results

All plasma samples were obtained from patients belonging to the MARS cohort admitted to ICUs. The MARS cohort includes patients above the age of 18, admitted to the ICUs of the UMC Utrecht, NL (study period 2011-present day) except for elective cardiothoracic surgical patients with an expected length of stay shorter than 24 h. For the current study cohort, we selected patients who had been admitted to the ICU at multiple occasions: the first; patients underwent a “routine” observation after elective surgery with generally an uncomplicated stay shorter than two days; the second and subsequent occasions were ICU-re-admissions after being suspected/diagnosed with sepsis or a septic shock.

**Figure 1:**
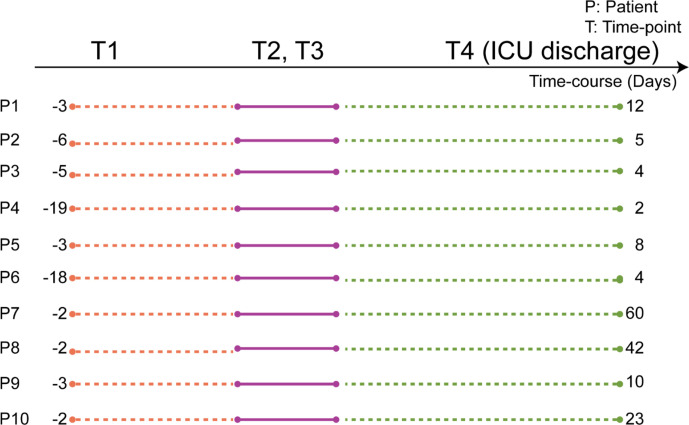
Scheme of the timing of the plasma sample collection, based on each patient’s physiological state. Plasma samples were collected from ten individuals each at four subsequent time-points. T1 is when each patient underwent elective surgery. The time-points T2 and T3 represent plasma collections at two consecutive days at the start of the assumed septic episode. T4 was collected when patients were discharged from the ICU. The numbers represent the elapsed number of days between the time points for each patient. Full patient descriptors including age and sex are provided in **Supplementary Table 2**.

For this study, we selected ten septic patients whose plasma samples had been collected longitudinally at four time-points (T1–T4). A schematic of the patient’s plasma collection details is depicted in **Figure 1**. Samples at T1 were obtained directly after elective surgery. T2 is at the start of the suspected septic episode, indicated by a substantial rise in the plasma abundance of the C-reactive protein (CRP). Plasma samples for T3 were collected the next day following T2. T4 was taken at discharge of the patients from the ICUs. There is a difference in the timing of plasma collection for each patient, as it was based on pathophysiological state, not on the exact number of days (see **Figure 1**). Notably, some patients were already released from the ICU after a few days (e.g. P3, P4, P6), while some stayed for a couple of weeks (e.g. P7, P8).

### Individual Plasma Proteome Profiles Confirm Known Trends Associated With Sepsis, Including α-1-Antichymotrypsin

Considering that sepsis is a multifactorial complex syndrome involving a multitude of cell types and organs, it comes to no surprise that clinical definitions of sepsis are primarily based on the patient’s symptoms instead of the underlying molecular signatures (48, 49). Several plasma and serum proteomics studies on sepsis have corroborated that the fundamental molecular mechanisms are indeed complex and diverse (14, 50–52).

**Figure 2:**
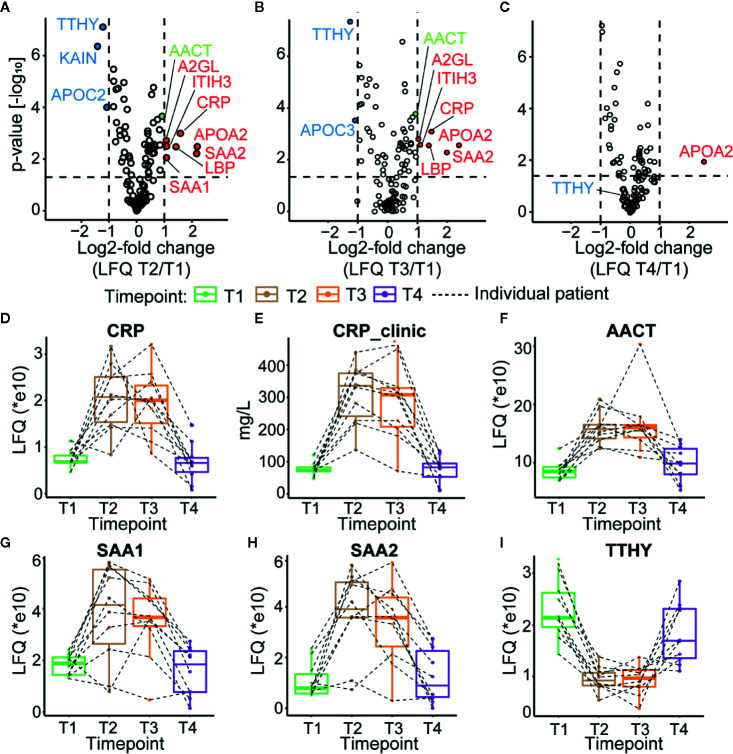
Plasma proteome profiles of septic patients. Volcano plots depicting the changes in protein abundances, normalized against T1, for T2 in **(A)**, T3 in **(B)**, and T4 in **(C)** averaged over all ten patients. Proteins increasing in abundance are depicted in red, while proteins becoming lower in abundance are shown in blue. AACT is depicted in green. Dotted lines indicate thresholds for significance and fold change. **(D)** Proteomics based quantitation of CRP levels per individual patient. **(E)** CRP levels measured in the clinic. **(F)** Proteomics based quantitation of AACT per individual patient. Proteomics based abundance levels for SAA1, SAA2, and TTHY in all individual patients are depicted in **(G–I)**, respectively. The depicted boxplots contain data for all patients across all four time-points. The dashed lines connect for each individual patient its corresponding time-points.

We first monitored the plasma proteome profiles of each septic patient, focusing just on the most abundant proteins. Using label-free quantitative (LFQ) proteomic profiling, we obtained abundance profiles of about the 200 most abundant plasma proteins (**Supplementary Table 3**). We used the LFQ values of the proteins in T1 to normalize a baseline for subsequent monitoring of the quantitative changes in the plasma proteome (**Supplementary Table 4**). Our analysis revealed about ten significantly higher abundant proteins at T2 and T3 compared to T1 **(** **Figures 2A, B** **)**. These upregulated proteins included several acute-phase response proteins (APPs) (ITIH3—inter-α-trypsin inhibitor, CRP, APOA2—apolipoprotein A2, SAA1—serum amyloid A1, SAA2—serum amyloid A2, A2GL—leucine-rich α-2-glycoprotein, and LBP—lipopolysaccharide-binding protein). These proteins display a wide range of functionalities, such as serine protease inhibitor (ITIH3), lipid transport (APOA2), and cholesterol transport (SAA1, SAA2). Both the upregulated and downregulated proteins observed in our analysis are commonly associated with sepsis, and other inflammatory physiologies, validating that all our patients indeed show the hallmarks of a septic episode (39, 53–56). Finally, following the discharge from the ICU patient plasma protein abundance profiles seemingly returned close to baseline (**Figure 2C**).

Since the clinical data on the patient of the MARS cohort contained information on the abundance level of CRP in plasma (**Supplementary Table 5**), we could compare the trends in CRP level per individual patient when measured with proteomics and ELISA (**Figures 2D, E**) and observed general agreement between these two methods. Additional examples of protein abundances of positive (SAA1 and SAA2) as well as negative (TTHY) acute phase proteins, as extracted from the proteomics data, are depicted in **Figures 2G–I**. The abundance trend of AACT, which we selected for further in-depth glycoproteoform profiling, partly due to its suggested active role in sepsis survivors (28), followed roughly a similar trend as CRP (**Figure 2F**), with an increase in abundance during sepsis in T2 and T3 and reverting close to baseline in T4 in all monitored patients.

As mentioned above, the plasma levels of CRP are often used to assess the condition of septic patients as one of the markers for potential release from the ICU. Our data show that several other APPs such as ITIH3, CRP, APOA2, SAA1, SAA2, A2GL, LBP, and AACT follow a similar trend.

### Alteration of Patients α-1-Antichymotrypsin Glycoproteoform Profiles During Sepsis

Our proteomics data revealed that the plasma concentration of AACT followed a similar trend as CRP and other APPs, with a notable increase in abundance upon sepsis, reverting to baseline at T4. Next, we questioned whether there would also be changes in the AACT glycoproteoform profiles of patients undergoing sepsis and whether such trends would be shared or unique for all studied patients. We chose to focus on AACT as it represents a plasma glycoprotein harboring some of the most elaborate glycosylation profiles, being modified by at least five diantennary type *N*-glycans (35, 36).

We and others have shown that high-resolution native mass spectrometry profiles of intact glycoproteins can provide valuable information about post-translational modifications (PTMs), but also another structural variability, such as genetic variants or mutations (26, 27, 40, 57, 58). Here we used a similar approach involving a rapid purification of AACT from plasma to investigate its proteoform profiles. Although AACT is one of the more abundant proteins in plasma (around 0.4 mg/L) (59), the high dynamic range of plasma protein levels hampers the facile isolation of AACT by ion exchange chromatography directly from plasma. We tackled this issue by first depleting the three most abundant plasma proteins (albumin, IgG, and serotransferrin) using a dedicated column and semi-automated set-up for immunodepletion, starting with 100 µL of a plasma sample. After the initial depletion, we subjected the eluate to ion-exchange chromatography (IEX) and collected the fraction containing AACT (**Supplementary Figure 2**). This fraction was subsequently directly analyzed by native MS.

Similar to the observations made by Wu et al. on acid glycoprotein (40) and Tamara et al. (27) on haptoglobin, the highly heterogeneous and complex AACT glycosylation profile precluded us from making a full spectral annotation. This high complexity is primarily caused by the high frequency and abundance of sialic acid (*N*-acetylneuraminic acid) moieties. They do not only expand the proteoform profiles immensely but also hamper the annotation of the glycan structures, as mass shifts caused by two fucosylation moieties or one sialylation moiety are hard to disentangle due to their alike masses (i.e., 291.1 and 292.1 Da, respectively). Hence, in line with the previous studies (27, 40), we first treated the AACT samples with sialidase to remove all sialic acid moieties prior to MS analysis, which indeed simplified the resulting native mass spectra (**Supplementary Figures 3A, B**). Sialidase treatment proved to be very reproducible (**Supplementary Figure 3C**). The deconvoluted native mass spectra of desialylated AACT from T1 of one donor (P10) contained around 30 distinct mass signals corresponding to various glycoproteoform variants (**Supplementary Table 6**). Most ion signals center around 55 kDa, whereas the “bare” AACT backbone mass is 45265.82 Da, leaving around 10 kDa of the measured mass corresponding to glycans. Notably, the ion signals of the non-desialyated AACT indicate a Mw of AACT of around 58 kDa, indicating that we eliminated on average 13–15 sialic acids using the sialidase treatment.

**Figure 3:**
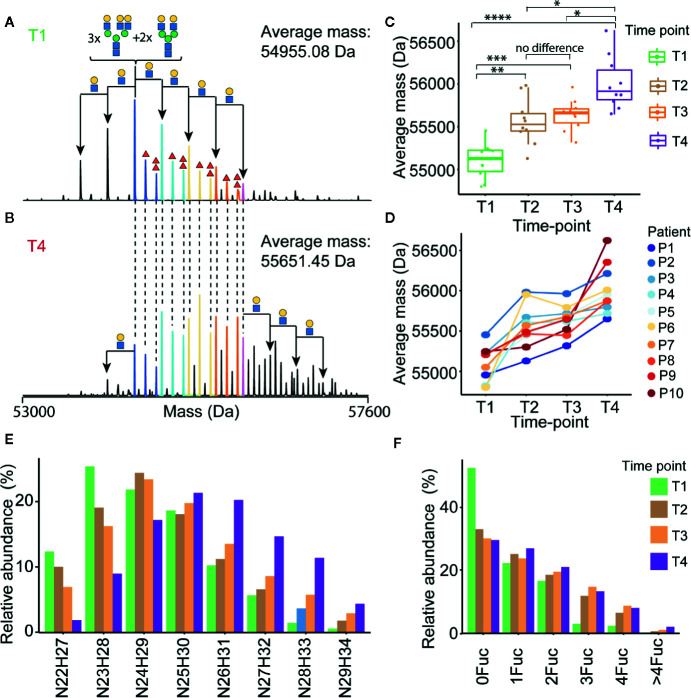
Alterations in α-1-antichymotrypsin glycosylation following sepsis prolong over time. Illustrative deconvoluted native mass spectra of desialylated AACT obtained at T1 and T4 are shown in **(A**, **B)**, respectively (for patient 1). The likely glycan compositions of the most abundant signals are annotated in **(A)**, whereby the HexNAcHex shifts from the most abundant peak are denoted with black arrows. Red triangles denote the presence of additional fucose moieties. Overlapping peaks in the spectra between **(A**, **B)** are color-coded, and the weighted average mass (WAM) is denoted in the upper right corner. WAMs for desialylated AACT for all patients and all time-points are shown in **(C)** represented in Boxplots and the * denote the p values from ANOVA analysis where *<0.05, **<0.01, ***<0.001, and ****<0.0005, and concomitant “spaghetti” plots connecting each patient’s data are depicted in **(D)**. Relative abundances of AACT glycoproteforms of patient P1 with the same HexNAcHex (N=HexNAc, H=Hex) or Fuc count, potentially distributed over 5 N-glycosylation sites, are depicted in **(E**, **F)**, respectively. An overview of the abundances of these glycoproteoforms in AACT for all other patients is provided in **Supplementary Figure 4**.

The most abundant signal in the desialyated AACT spectrum corresponds to the AACT protein modified with five *N*-glycans. Using mass matching, we deduced a likely composition of these five glycans and achieve full annotation of the recorded proteoform profiles as depicted in **Figure 3A**. To further support our annotations, we performed a glycopeptide analysis of all times points from patient 10. Although such analysis is challenging due to the poor accessibility of AACT glycosylation sites, we managed to cover three out of six potential N-glycosylation sites. **Supplementary Figure 2** shows site-specific extracted ion-chromatogram-based relative quantification of sites N106, N127, and N271. The data provides evidence that AACT contains a complex type of N-glycans and that the branching and fucosylation are changing within the four-time points. We continued to investigate these changes for all patients with the analysis of native MS proteoform profiles. By comparing the annotated native MS profiles of T1, T2 and T3, we see a substantial shift in the distribution towards higher masses caused by increased fucosylation and additional branching/elongation of the *N*-glycans (**Supplementary Figure 4** and **Supplementary Table 6**). Surprisingly, this glycan remodeling (that is increase in fucosylation and/or branching/elongation) continues even further onto T4, where we reproducibly observed an even higher degree of AACT glycosylation (**Figures 3B–F** and **Supplementary Figure 5**). Since this extensive glycan remodeling, with the enhanced occurrence of fucosylation and additional branching/elongation, increases the glycoproteoform mass, we decided to determine the weighted average mass (WAM) of AACT to effectively and quantitatively visualize these mass shifts. This average mass is a single value representing the WAM of all annotated AACT glycoproteoforms (see *Methods* section for parameters and annotation of native mass spectra). Using this concept, it becomes immediately apparent that across all patients, we observe a substantial initial glycan remodeling in T2 and T3 when compared to T1 (**Figures 3C, D** and **Supplementary Table 7**). Additionally, in contrast to the observation that the AACT protein abundance returns at T4 to baseline levels comparable to T1 (**Figure 2F**), we observed a prolonged increase in glycan extensions at T4 when compared to all earlier three time points in all individual patients’ AACT (**Figures 3C, D** and **Supplementary Table 6**). From this data it appears that an acute inflammatory event, in this case sepsis, induces a longer-term glycosylation remodeling on AACT, even though that the abundance levels of AACT (and CRP) return to baseline levels and the patients have been released from the ICU. Sepsis seems to leave a longer-lasting imprint on the glycoproteoform profile of plasma AACT.

Despite an evident trend in the increased WAM of AACT across four-time points observed in all patients, we observed significant differences in glycoproteoform profiles between the different patients, which indicated that the T1 baseline glycoproteoform profile for each patient is already quite different. This variability becomes evident in **Figure 3D**, wherein we plotted the trends for each patient separately. For instance, although both P1 and P2 show a similar continuous trend in the mass increase over time, the end-point in WAM for P1 (i.e., at T4) is similar to the WAM for P2 at the start (i.e., at T1). Such trends would not be possible to observe if we had pooled all ten samples, highlighting the need for more personalized longitudinal glycoproteoform profiling.

### Personalized Glycoproteoform Profiling to Assess Inter-Individual Glycosylation Heterogeneity

**Figure 4:**
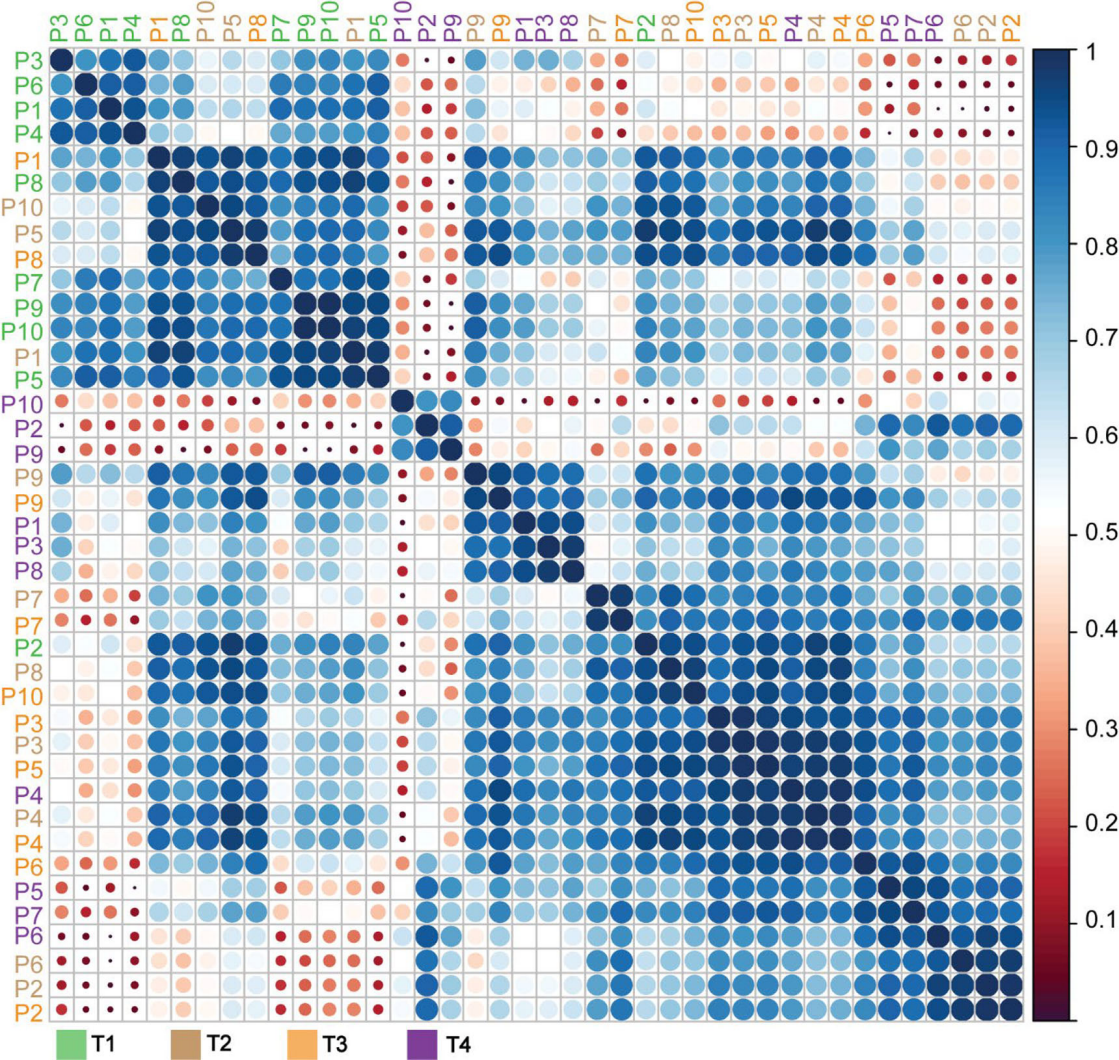
Correlation matrix of α-1-antichymotryspin glycoproteoform profiles for all patients at all time points. Native mass spectra for each patient and time-points (10x4 spectra) were correlated with each other and clustered based on the derived Pearson correlation. Patients are color-coded according to the legend below the matrix. The extracted correlation is indicated both by the scale bar on the right, with dark blue representing the highest correlation, and the size of the circles, whereby bigger circles represent higher correlations than smaller circles.

To further assess inter-individual glycosylation heterogeneity in AACT, we used a “spectral barcoding” approach (57) wherein the deconvoluted native mass spectra of AACT from each patient and each time-point are used as glycoproteoform signatures (i.e. barcode). These signatures, consisting of deconvoluted mass peaks and intensities, are subsequently directly correlated with each other for mass spectral similarity, providing a Pearson correlation coefficient. As entries, we have the 40 mass spectra (from 10 patients at four-time points) and calculated the correlation coefficient between them. In **Figure 4** an overview of all this data is given in the form of a correlation matrix, whereby spectra that are alike (having a high Pearson correlation) cluster together. From the upper left quadrant of this matrix, it is apparent that the spectra of all patients at T1 cluster well together, with notable exceptions (e.g. P2). However, except for some samples taken at T4, i.e. from patients P10, P2, and P9, which show a distinctive cluster, we did not observe a clear separation at any of the other time-points. This representation corroborates the observation that each patient has a unique baseline in their AACT glycoproteoform profiles, and the onset of sepsis causes extensive remodeling of the AACT glycoproteoform profile. Intrigued by this observation of continuous glycosylation remodeling even up to T4 (when patients have already been released from the ICU, and AACT and CRP levels have returned to “normal”), we focused next solely on the T1 and T4 “extreme” samples.

**Figure 5:**
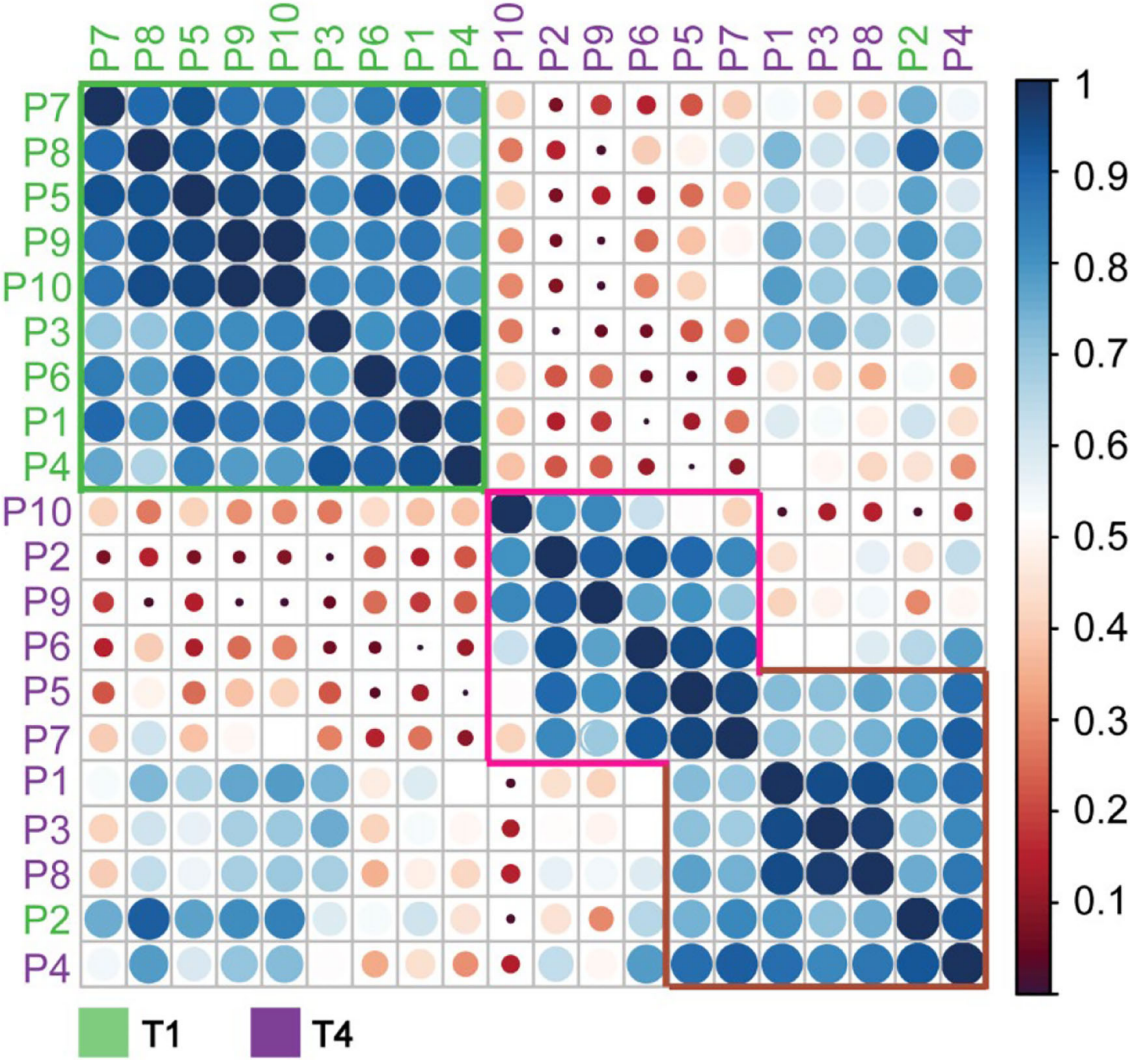
Correlation matrix of α-1-antichymotryspin glycoproteoform profiles for all individual patients at time points T1 and T4. Native mass spectra for each patient and time-points were correlated with each other and clustered based on the Pearson correlation. Patients are color-coded according to the time-point with green corresponding to T1 and red to T4. Highlighted are three distinctive clusters coming out of this analysis: green, purple, and brown. The most likely feature distinguishing these clusters is the average weighted masses, which increase for the data from the green box, *via* the brown to the pink box.

Constructing a similar correlation matrix by taking only the data of the patients at T1 and T4 into account, we observed a clear separation between those two-time points (**Figure 5** **).** We again observed one notable exception, T1 of patient 2, which seemingly falls into the late-time point cluster. Notably, patient 2 already exhibited already a degree of glycosylation, and thus highest WAM at T1, as depicted in **Figures 3C, D**. Additionally, we noticed that the spectra of all patients taken at T4 were separated into two partially overlapping clusters, indicated in **Figure 5** with a pink and brown box, respectively. We first assessed whether this separation might be due to the differences in the sample collection points of T4 (see **Figure 1**) (e.g. T4 for patient 4 was four days after T3, while for patient 7 this time difference was 60 days), but this turned out not to be a determining factor (**Figure 5** **)**. The most evident factor correlating to the distinction between the data in the purple and brown boxes was the extent of glycan remodeling at T4, as also represented by the increase in WAM values. The patients in the brown (low-remodeling) cluster display a lower increase in WAM when compared to those in the purple box (high-remodeling). This difference means that plasma AACT, especially from patients in this purple box, becomes extensively remodeled due to increased fucosylation and glycan branching/LacNAc elongation. In agreement, AACT glycoproteoform profiles from the T4 samples of patients in the brown box also correlate better with those in cluster 1, as they have become remodeled to a lesser extent.

**Figure 6:**
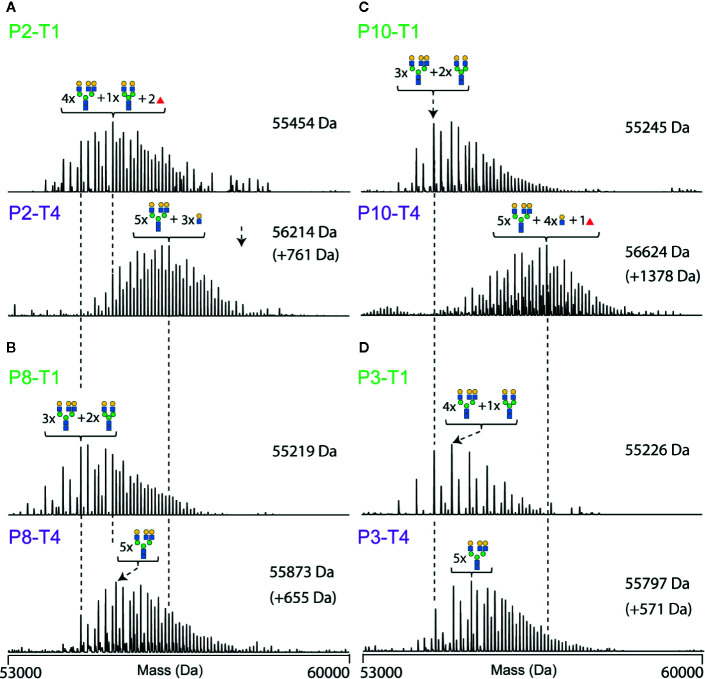
Deconvoluted native mass spectra of desialylated AACT, illustrating unique differences in between patients and the extensive glycoproteoform remodeling observed between T1 and T4. Spectra are shown for four patients, each at two-time points (T1 at the top and T4 at the bottom). Data for patient P2 are shown in **(A)**, P8 in **(B)**, P10 in **(C)**, and P3 in **(D)**. The most abundant peak in each spectrum is annotated with a likely glycan composition. As a visual guide, dashed lines connect peaks of the same mass.

To further illustrate the inter-individual diversity in AACT glycoproteome profiles, representative deconvoluted native mass spectra of selected samples (T1 and T4 pairs) are depicted in **Figure 6**. For instance, patient 2 exhibits already a relatively complex and high mass glycoproteoform profile at T1, similar to the profiles observed at T4 for some of the other patients (**Figure 6**). Also, the extent of glycan remodeling can vary considerably, whereby especially P10 shows a very high level of remodeling (**Figure 6**). This data demonstrates that each person exhibits their unique glycoproteome profile.

## Discussion

**Figure 7:**
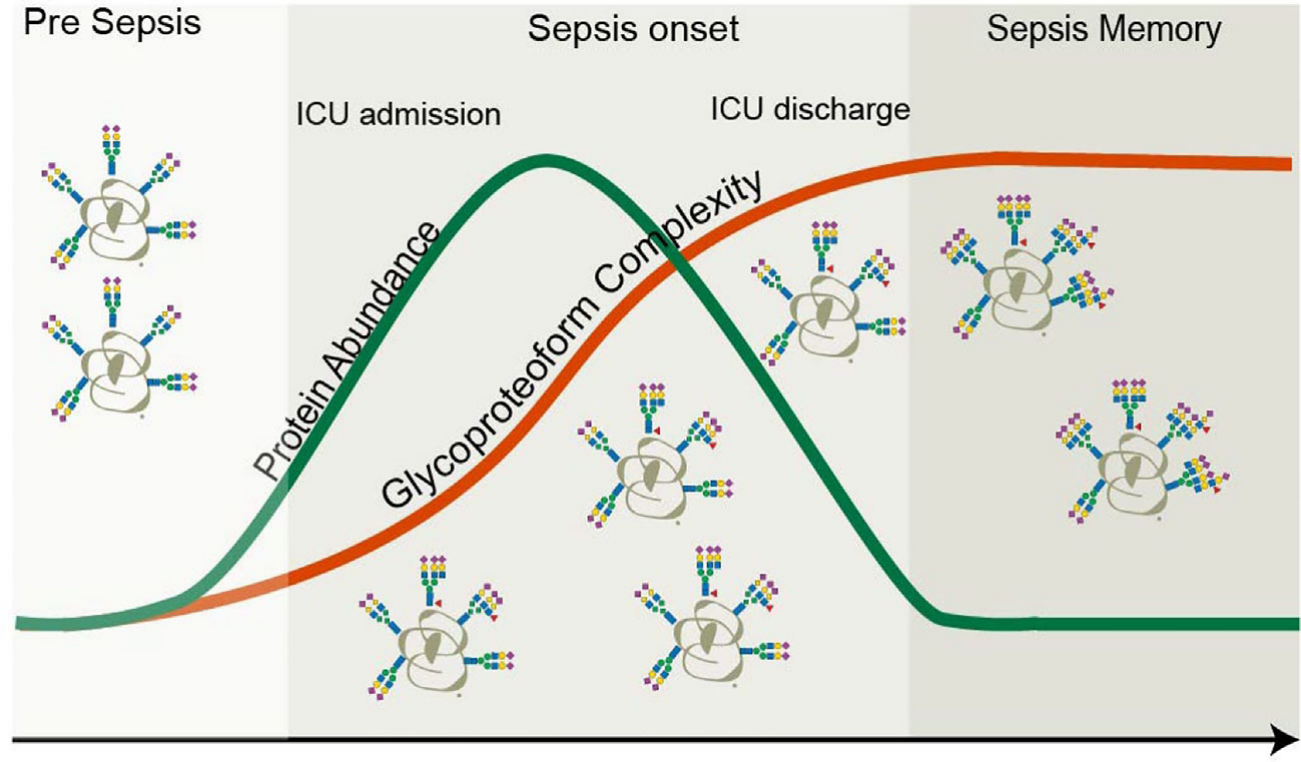
Protein *versus* proteoform profiling. In this study, both the protein abundance as well as proteoform profile of the plasma protein AACT were monitored longitudinally in ten patients that experienced a septic episode. As AACT is a positive acute-phase protein, its concentration increased during the septic episode and returned to baseline, following the same trend in time as the clinically used marker CRP. Upon return to baseline of CRP (and thus also AACT), patients are typically released from the intensive care unit. At the start of the septic episode also the glycoproteoform profile of AACT started to change and become remodeled and more complicated by the accumulation of additional branches, LacNAc elongations, and additional fucose moieties. Glycosylation remodeling proceeds even after the protein levels have gone back to “normal,” and patients are released from the ICU. This finding means that a memory effect of the septic episode is still present in the patients’ AACT days to weeks after the release from the ICU.

In this study, we investigated by proteomics approaches small amounts of plasma obtained from ten patients longitudinally at four time-points throughout a non-lethal septic episode. We first confirmed that this acute inflammatory event resulted in the temporally higher plasma abundance of several positive acute-phase proteins (APPs) that returned to normal levels following partial recovery, whereby patients were released from the ICU. These proteins included the often-used clinical marker CRP, but also, for instance, SAA1, SAA2, and AACT. Next, we obtained by native mass spectrometry the glycoproteoform profiles of one of these proteins, AACT. We focused on AACT as a previous study demonstrated the upregulation of AACT glycopeptides especially in sepsis survivors (28). In our study, we observed a substantial remodeling in its glycosylation already at the onset of the septic episode. Remarkably, this remodeling prolonged even much further, extending beyond the time-point of release of the patients from the ICU. These changes in AACT glycosylation originate primarily from continuous increases in *N*-glycan branching and additional fucosylation. The observed prolonged remodeling of the glycosylation of AACT is thus in sharp contrast to the trend observed for the plasma abundance of AACT, which reverted to “normal” at T4, i.e., when patients were released from the ICUs (**Figure 7**).

It is well-established that an inflammatory event, regulated by cytokines (predominantly IL-6), can cause differential glycosylation processing during glycoprotein synthesis in the liver (60–62). It is important to note that glycan remodeling can occur independently of protein synthesis, functionally decoupling protein from glycoproteoform abundance levels as we indeed here observe for plasma AACT. Our findings here on AACT also are in line with early observations reported on the α-1-acid glycoprotein. These studies showed that acute inflammation induces action of inflammatory cytokines affecting the expression of the α1-3 fucosyltransferase enzyme in the liver, resulting in increased levels of sialyl-Lewis X on the α-1-acid glycoprotein (63–65).

One possible reason for the here-observed extensive glycosylation remodeling might be a defense mechanism to protect the patient against immune over-reaction. For instance, aberrant leukocyte activation and recruitment in host tissues can result in the breakdown of the vascular endothelium, organ failure, and death (66, 67). Since sialyl-Lewis X and E-selectin mediate leukocyte extravasation, expression of the same epitope on APPs might represent a way to partially inhibit such interactions (63).

Concerning the observed increase in AACT fucosylation, we hypothesize that these fucose moieties are situated in the antennae of the glycans rather than being attached to the chitobiose core, based on the facts that APPs are seldom core-fucosylated (68, 69) and AACT has been reported to carry sialyl-Lewis X motif (70). This location of the fucose moieties is in line with the observed concomitant increase in *N*-glycan branching, which results in many more acceptor sites for antennary fucosylation, thus increasing the avidity of sialyl-Lewis X epitope presentation. Although the functional role of *N*-glycosylation in AACT is still elusive, there are a few studies reported on the somewhat similar plasma serine protease, alpha-1-antitrypsin (AAT) (61, 71). *N*-Glycans decorating ATT have a multifaceted function and play a role in protecting the protein from proteolysis, preventing aggregation as well as glycoform-glycoform dependent interaction with human neutrophil elastase (72–74). Even if this would also be true for AACT, it is unlikely that the glycosylation changes of AACT during sepsis progression observed here influence these potential functions of AACT glycans in particular. Nevertheless, the significant changes in glycoproteoform profiles of AACT could have an impact on its interaction properties with substrates and other proteins. This effect would not be unprecedented as Wu et al. and Tamara et al. described that *N*-glycan heterogeneity might regulate interactions between plasma proteins, notably, between haptoglobin and hemoglobin (27, 40).

In conclusion, we demonstrate that monitoring, at high-resolution, proteoform profiles of individual glycoproteins, such as AACT, provides valuable personalized information complementing traditional measurements of plasma concentrations of APPs for monitoring sepsis progression. Focusing on just this single protein, we reveal that each person’s AACT glycoproteform profiles are unique. We expect this to be true for many other plasma glycoproteins, as corroborated by our recent study on plasma fetuin, showing that its glycoproteoform profiles are affected not only by physiological states but also by frequent genetic polymorphism (26). APPs represent a group of proteins in which concentration levels are highly responsive to inflammatory events during sepsis and other diseases. Our findings on AACT suggest that next to the rate of synthesis of these proteins, we should be critical of their structural changes and dynamics since these are unique in patients and may provide a deeper understanding of complex pathologies such as sepsis.

## Data Availability Statement

The datasets presented in this study can be found in online repositories. The names of the repository/repositories and accession number(s) can be found below: https://massive.ucsd.edu/ProteoSAFe/static/massive.jsp, MSV000086041.

## Ethics Statement

The studies involving human participants were reviewed and approved by the UMCU institutional review board who approved an opt-out method for consent (protocol numbers 10-056C/18-192). The patients/participants provided their written informed consent to participate in this study.

## Author Contributions

TČ, Y-HL, VF, and AH designed the research and planned the experiments. TČ, Y-HL, and VF performed all the experiments and data analyses. TČ, VF, and AH wrote the original draft, which was read, improved, and approved by all co-authors. KR contributed to the study design. MV, MB, and OC provided the MARS sepsis cohort samples and selected the patient population used in this study. All authors contributed to the article and approved the submitted version.

## Conflict of Interest

The authors declare that the research was conducted in the absence of any commercial or financial relationships that could be construed as a potential conflict of interest.
