# Supplementary material for: Glycoproteoform Profiles of Individual Patients’ Plasma Alpha-1-Antichymotrypsin are Unique and Extensively Remodeled Following a Septic Episode
Source: Front Immunol. 2021 Jan 14;11:608466. doi: 10.3389/fimmu.2020.608466 (PMC7840657; doi:10.3389/fimmu.2020.608466)
Supplement: Supplementary file 2 [file DataSheet_2.docx]

**Supplementary Figures**


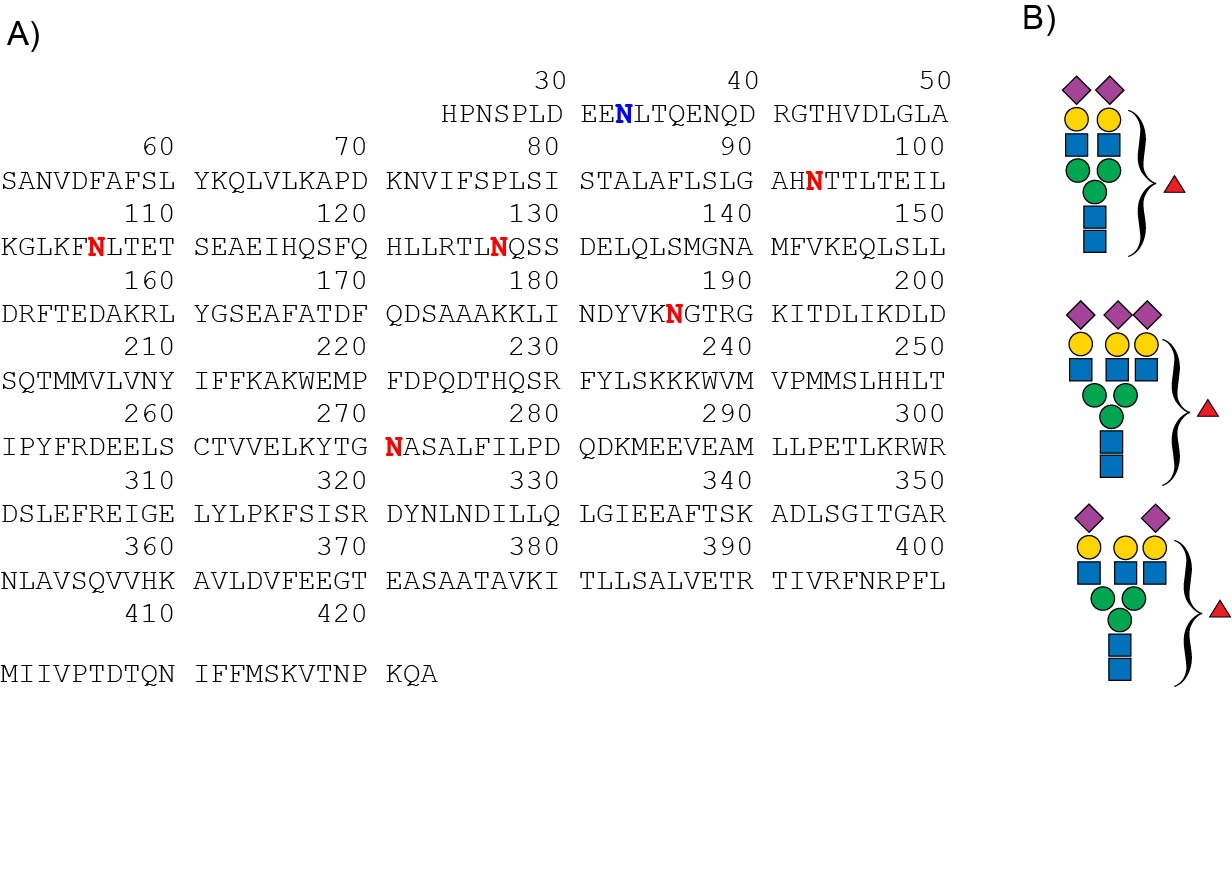


**Figure S1. The amino acid sequence of AACT with reported N-glycosylation sites. A)** amino acid sequence of AACT with the reported highly occupied N-glycosylation sites highlighted in red, and the low occupancy site highlighted in blue(1, 2). **B)** Scheme of the most abundant AACT glycoforms reported in the literature(2, 3).


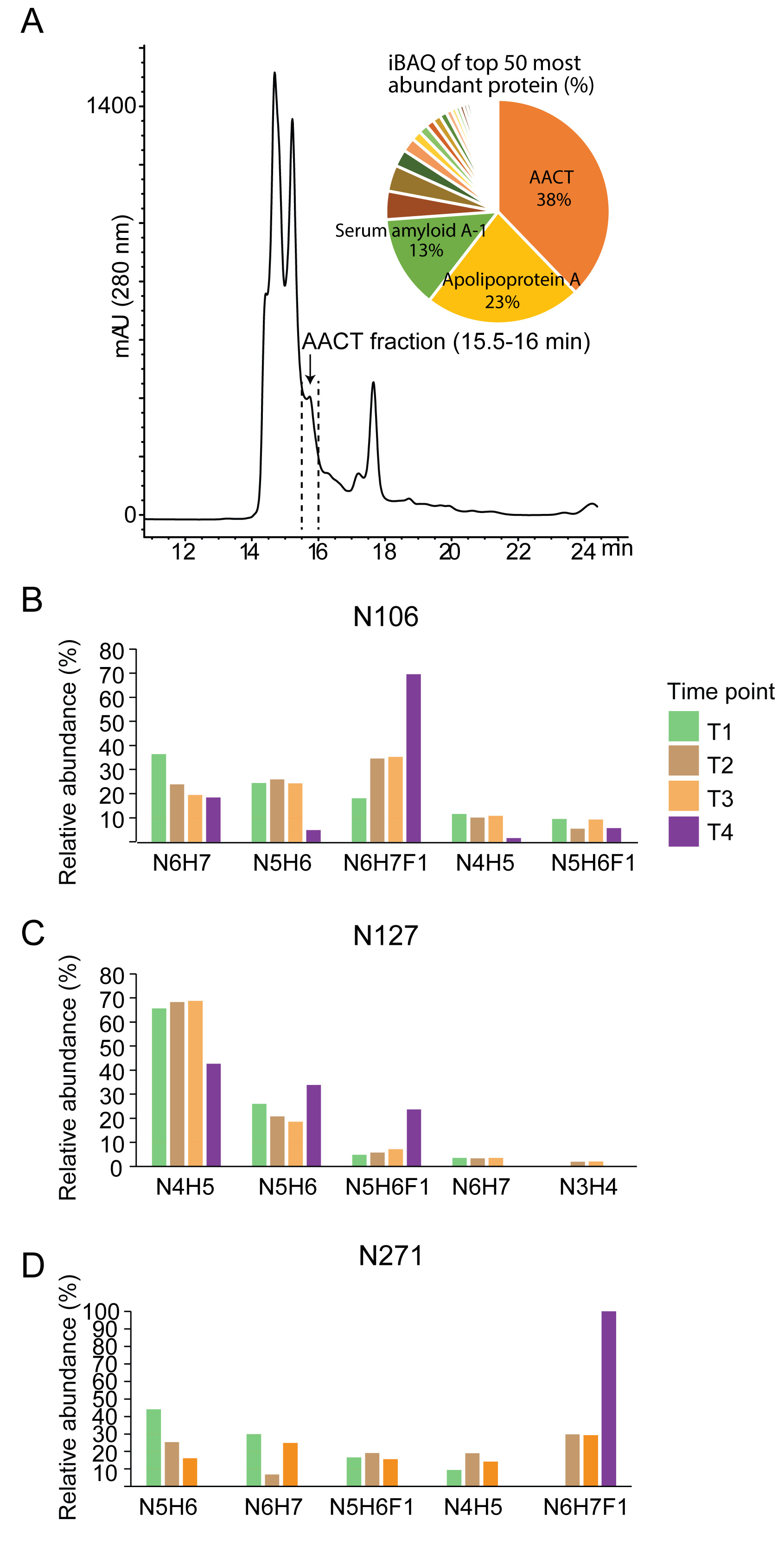


**Figure S2. Ion Exchange Chromatography separation and glycopeptide analysis of AACT.**  A) An example of a plasma ion-exchange chromatogram is shown, whereby the collected fraction containing AACT is indicated and the corresponding iBAQ intensities from the fraction are shown. B-D) Intact glycopeptide analysis of P10 T1-4 covering N106, N127 and N271 glycosylation sites.

**
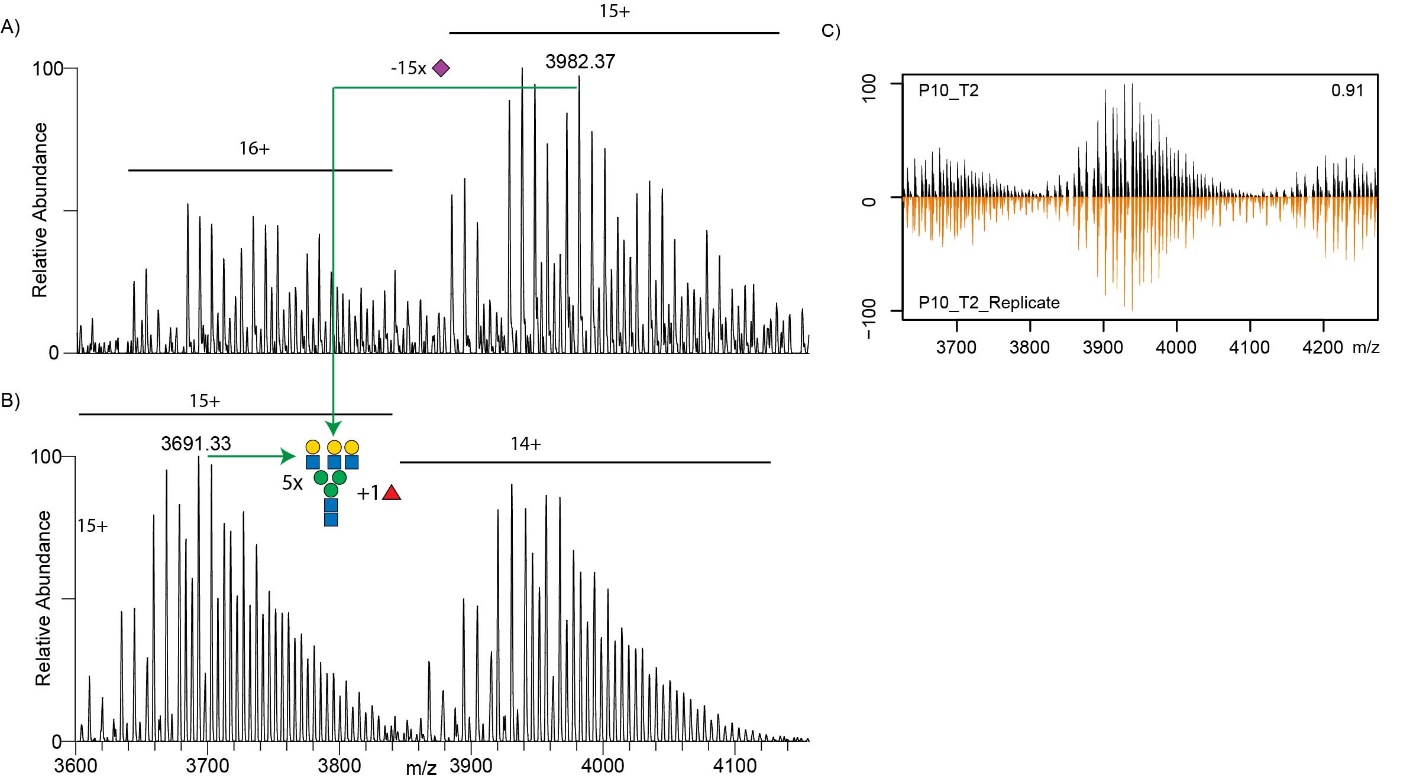
Figure S3. Clarification of glycoproteoform profile of AACT by sialidase treatment. A)** Native mass spectrum of sialylated AACT **B)** Native mass spectrum of sialidase treated AACT in **B)**. Horizontal lines denote the charge state, and the green arrows connect the glycan composition corresponding to three triantennary N-glycans and fucose with 15 sialic acids. Loss of 15 sialic acids can be observed in the sialidase treated spectrum. **C)** Technical reproducibility of the sialidase treatment represented by native MS measurements of two samples of sialidase treated samples of P10 of T2. On top, T2 of P10 is shown. Below in orange, a replicate measurement for the same patient time-point is shown. The upper right corner shows the high Person correlation score (i.e., 0.91).


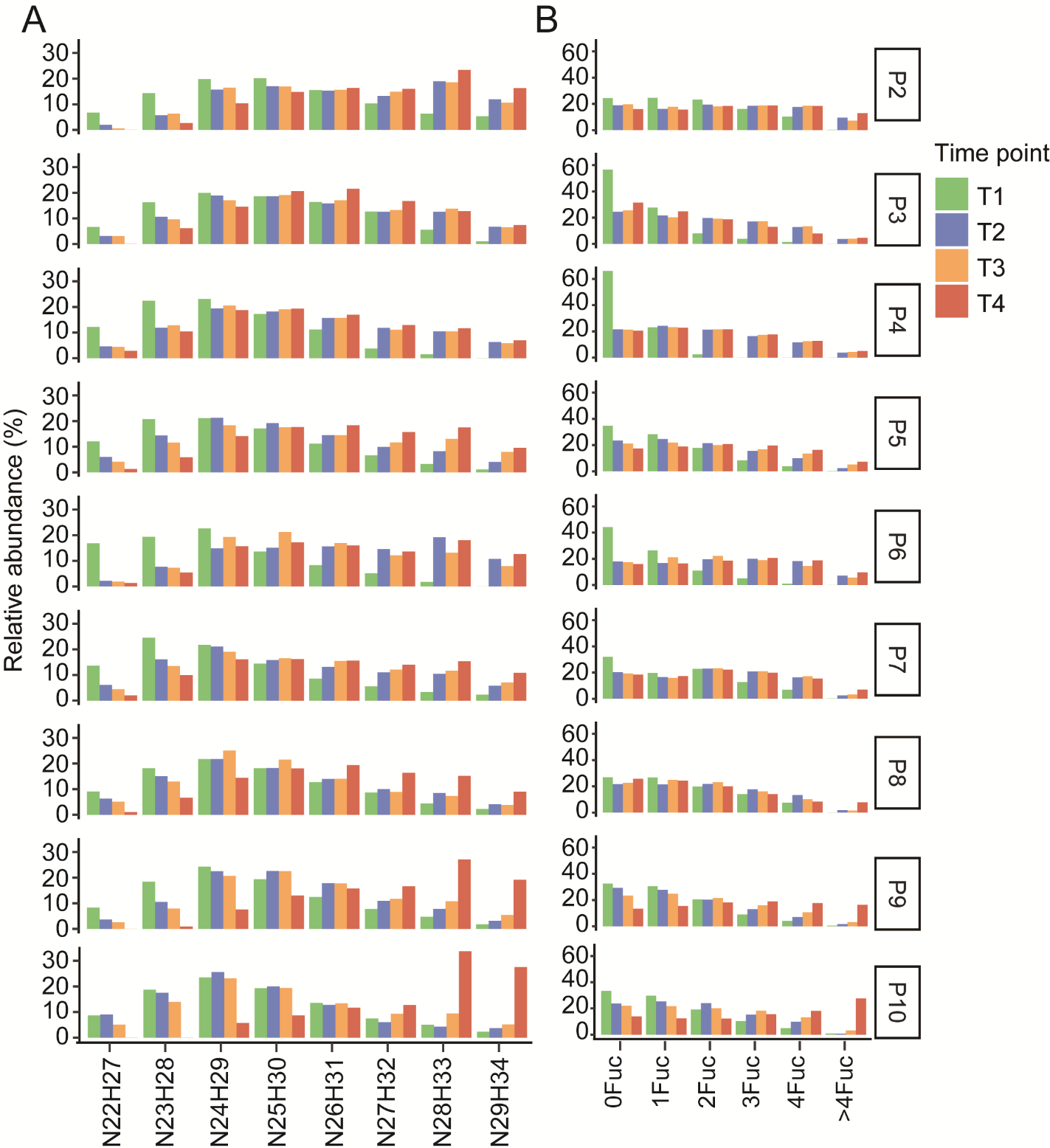


**Figure S4. Changes in the level of branching and fucosylation of AACT across the four time points for each patient. A)** Glycoproteoform compositions with the same HexNAcHex (N=HexNAc, H=Hex) composition were summed up together and plotted for all four-time points **B)** All glycoproteoform compositions containing at least one fucose were summed up and sorted by their fucose content. Of note fucoses can be spread across all 5 N-glycan.


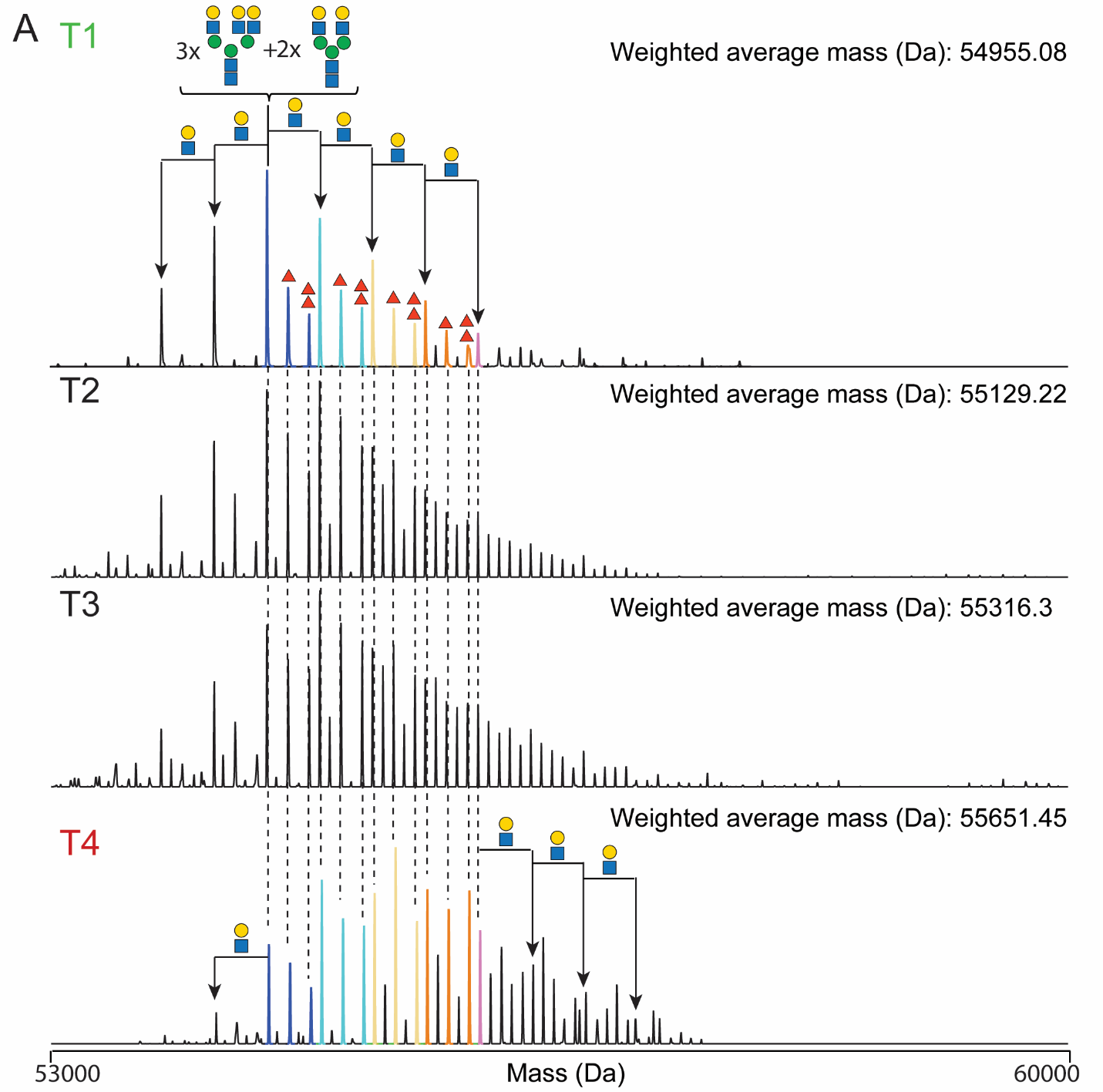


**Figure S5. Illustrative deconvoluted native mass spectra displaying the continuous remodeling of alpha-1-antichymotrypsin glycosylation across the four-time points as here depicted for patient P1.**

**REFERENCES**

1. York, W. S., Mazumder, R., Ranzinger, R., Edwards, N., Kahsay, R., Aoki-Kinoshita, K. F., Campbell, M. P., Cummings, R. D., Feizi, T., Martin, M., Natale, D. A., Packer, N. H., Woods, R. J., Agarwal, G., Arpinar, S., Bhat, S., Blake, J., Castro, L. J. G., Fochtman, B., Gildersleeve, J., Goldman, R., Holmes, X., Jain, V., Kulkarni, S., Mahadik, R., Mehta, A., Mousavi, R., Nakarakommula, S., Navelkar, R., Pattabiraman, N., Pierce, M. J., Ross, K., Vasudev, P., Vora, J., Williamson, T., and Zhang, W. (2020) GlyGen: Computational and Informatics Resources for Glycoscience. *Glycobiology* 30, 72–73

2. Sun, S., Hu, Y., Jia, L., Eshghi, S. T., Liu, Y., Shah, P., and Zhang, H. (2018) Site-Specific Profiling of Serum Glycoproteins Using N-Linked Glycan and Glycosite Analysis Revealing Atypical N-Glycosylation Sites on Albumin and α-1B-Glycoprotein. *Anal. Chem.* 90, 6292–6299

3. LAINE, A., HACHULLA, E., STRECKER, G., MICHALSKI, J.-C., and WIERUSZESKI, J.-M. (1991) Structure determination of the glycans of human-serum alpha1-antichymotrypsin using 1H-NMR spectroscopy and deglycosylation by N-glycanase. *Eur. J. Biochem.* 197, 209–215
